# Gene expression profiling of the human natural killer cell response to Fc receptor activation: unique enhancement in the presence of interleukin-12

**DOI:** 10.1186/s12920-015-0142-9

**Published:** 2015-10-15

**Authors:** Amanda R. Campbell, Kelly Regan, Neela Bhave, Arka Pattanayak, Robin Parihar, Andrew R. Stiff, Prashant Trikha, Steven D. Scoville, Sandya Liyanarachchi, Sri Vidya Kondadasula, Omkar Lele, Ramana Davuluri, Philip R. O. Payne, William E. Carson

**Affiliations:** The Arthur G. James Comprehensive Cancer Center and Solove Research Institute, The Ohio State University, Columbus, OH 43210 USA; Medical Scientist Training Program and Biomedical Sciences Graduate Program, The Ohio State University, Columbus, OH 43210 USA; Department of Biomedical Informatics, The Ohio State University, Columbus, OH 43210 USA; Department of Pediatrics, The Cleveland Clinic, Cleveland, OH 44106 USA; Division of Human Cancer Genetics, The Ohio State University, Columbus, OH 43210 USA; Departments of Oncology and Medicine, Wayne State University and Barbara Ann Karmanos Cancer Institute, Detroit, MI 48201 USA; Robert H. Lurie Comprehensive Cancer Center, Northwestern University, Chicago, IL 60611 USA; Department of Surgery, The Ohio State University, Columbus, OH 43210 USA; The Ohio State University College of Medicine, N924 Doan Hall, 410 West 10th Ave., Columbus, OH 43210 USA

**Keywords:** NK cells, CD16, Gene microarray, Interleukin-12, Interferon-gamma

## Abstract

**Background:**

Traditionally, the CD56^dim^CD16^+^ subset of Natural Killer (NK) cells has been thought to mediate cellular cytotoxicity with modest cytokine secretion capacity. However, studies have suggested that this subset may exert a more diverse array of immunological functions. There exists a lack of well-developed functional models to describe the behavior of activated NK cells, and the interactions between signaling pathways that facilitate effector functions are not well understood. In the present study, a combination of genome-wide microarray analyses and systems-level bioinformatics approaches were utilized to elucidate the transcriptional landscape of NK cells activated via interactions with antibody-coated targets in the presence of interleukin-12 (IL-12).

**Methods:**

We conducted differential gene expression analysis of CD56^dim^CD16^+^ NK cells following FcR stimulation in the presence or absence of IL-12. Next, we functionally characterized gene sets according to patterns of gene expression and validated representative genes using RT-PCR. IPA was utilized for biological pathway analysis, and an enriched network of interacting genes was generated using GeneMANIA. Furthermore, PAJEK and the HITS algorithm were employed to identify important genes in the network according to betweeness centrality, hub, and authority node metrics.

**Results:**

Analyses revealed that CD56^dim^CD16^+^ NK cells co-stimulated via the Fc receptor (FcR) and IL-12R led to the expression of a unique set of genes, including genes encoding cytotoxicity receptors, apoptotic proteins, intracellular signaling molecules, and cytokines that may mediate enhanced cytotoxicity and interactions with other immune cells within inflammatory tissues. Network analyses identified a novel set of connected key players, *BATF, IRF4, TBX21*, and *IFNG*, within an integrated network composed of differentially expressed genes in NK cells stimulated by various conditions (immobilized IgG, IL-12, or the combination of IgG and IL-12).

**Conclusions:**

These results are the first to address the global mechanisms by which NK cells mediate their biological functions when encountering antibody-coated targets within inflammatory sites. Moreover, this study has identified a set of high-priority targets for subsequent investigation into strategies to combat cancer by enhancing the anti-tumor activity of CD56^dim^CD16^+^ NK cells.

**Electronic supplementary material:**

The online version of this article (doi:10.1186/s12920-015-0142-9) contains supplementary material, which is available to authorized users.

## Background

Natural killer (NK) cells are large granular lymphocytes that participate in the innate immune response to virally infected and malignant cells. NK cells possess a diverse repertoire of natural killer cell receptors (NKRs) that allow NK cells to recognize MHC^low^ or MHC^negative^ target cells or non-classical MHC molecules (e.g. MICA/B and ULBP). The physiological functions of NK cells are regulated by a delicate balance of signals transmitted via activating and inhibitory NKRs [1]. NK cells interact with antibody (Ab)-coated target cells via the FcγRIIIa or CD16, an activating, low-affinity receptor for the Fc portion of IgG [2]. Further, our group has demonstrated *in vitro* and *in vivo* that co-stimulation of NK cells with stimulatory cytokines such as interleukin (IL)-12 significantly enhances the immune response to Ab-coated tumor cells [3]. NK cells are uniquely equipped to mediate such Ab-dependent effector functions because they contain abundant cytolytic granules, prominently express cellular adhesion molecules, constitutively express multiple cytokine receptors, and rapidly secrete immune modulatory cytokines following activation. These properties provide NK cells with the ability to directly lyse cellular targets as well as coordinate the developing adaptive immune response.

Human peripheral blood NK cells may be divided into two subsets based on their cell surface density of CD56 and CD16 molecules. The majority of NK cells (approximately 90 %) are phenotypically characterized as CD56^dim^CD16^+^, while the remaining cells are CD56^bright^CD16^neg^ [4]. The CD56^bright^CD16^neg^ NK cell population is thought to be the immediate precursor to the CD56^dim^CD16^+^ subset and plays an important role in regulating immune responses via cytokine-mediated cross-talk with T cells and dendritic cells (DCs) [5–7]. In contrast, the cytotoxic CD56^dim^CD16^+^ NK cell subset expresses higher levels of chemokine receptors, and therefore is preferentially recruited to peripheral sites of inflammation [8]. Within inflammatory environments, encounters between CD56^dim^CD16^+^ NK cells and target cells as well as exposure to locally secreted inflammatory cytokines promotes activation of this subset, leading to dramatically increased cytotoxic activity against target cells and abundant pro-inflammatory cytokine production equivalent to that of the CD56^bright^CD16^neg^ population [9–11]. The early recruitment and activation of CD56^dim^CD16^+^ NK cells to sites of inflammation raises important questions regarding the potential immune functions of these cells that extend beyond their cytotoxic capabilities. Thus, the present study has sought to elucidate the complex genomic profile of activated CD56^dim^CD16^+^ NK cells via a series of laboratory and bioinformatics-based approaches.

**Fig. 1:**
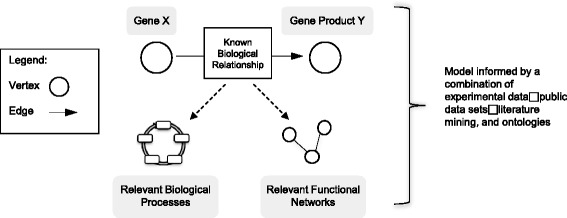
Overview of the network-level systems analysis approach. This type of methodology utilizes a combination of observed data, public data sets, the mining of applicable domain literature, and/or ontologies (e.g., expertly curated collections of domain knowledge represented in a computable format). In this example, vertices (e.g., genes, gene products, and biological structures or functions) are linked together by edges that represent relevant biological relationships

The systems-level bioinformatics-based approaches employed in this study build upon the results of our laboratory-based studies, augmented with publicly available data sets and knowledge collections. Specifically, we have applied network-based analysis methods to gene expression data derived from microarray analyses. In such analyses, individual biomolecular entities (e.g., genes, gene products, bio-chemical agents, etc.) belong to a larger system, with specific structural or functional relationships serving to “link” together the entities comprising that system. In these systems, the biomolecular entities may be referred to as “vertices” and the relationships that connect those vertices may be referred to as “edges” [12]. These relationships are identified via canonical information retrieval workflows. Such workflows are designed for inspection of multiple sources of relevant data; including but not limited to biomedical literature, public data sets, and collections of knowledge structured in formalized constructs known as ontologies. A simplified and illustrative example of this type of network-level systems analysis approach is provided in Fig. 1. Once a network construct is generated, it may be used to identify critical vertices that, if targeted in a diagnostic or therapeutic context, have a maximal ability to influence the function of the overall biological system in question. Indeed, it has been described that such network-based approaches may enhance the ability to identify “high yield” targets for diagnostics or therapeutics, thus optimizing the selection and pursuit of actionable and clinically relevant hypotheses [13]. To our knowledge, this is the first network analysis approach used to identify a set of high-priority gene targets based on transcriptome profiling of NK cells under unique stimulation conditions.

In this study, we present a novel application of a systems biology approach to evaluate the global patterns of gene expression in CD56^dim^CD16^+^ NK cells following FcR stimulation in the presence or absence of the potent immune-stimulatory cytokine, IL-12. Oligonucleotide microarrays and Real-Time PCR were utilized to elucidate expression patterns of various genes in activated NK cells. Bioinformatics-based approaches were employed to build upon our observations and inform future investigation regarding the role of activated CD56^dim^CD16^+^ NK cells. Further, the immunologic and bioinformatics-based analyses of NK cell function in response to clinically relevant co-stimuli presented herein may be useful for the evaluation of experimental immunologic results in future studies. Importantly, the transcriptional profiles described in this manuscript provide insight regarding the potential functions of this NK cell subset during interactions with Ab-coated targets in the context of host defense as well as in the setting of monoclonal antibody therapy for cancer.

## Methods

### Cytokines and antibodies

Recombinant human IL-12 (rhuIL-12) was provided by Genetics Institute, Inc. (Cambridge, MA). Anti-FcγRIIIa antibody 3 g8 was obtained from Medarex Inc. (Annandale, NJ). Polyclonal human IgG (huIgG) was purchased from Sigma-Aldrich Co. (St. Louis, MO).

### Isolation of human NK cells

NK cells were isolated directly from fresh peripheral blood Leukopaks (American Red Cross, Columbus, OH) by 30-min incubation with RossetteSep cocktail (Stem Cell Technologies, Vancouver, BC), followed by ficoll hypaque density gradient centrifugation. NK cells were > 95 % CD56^+^ by FACS analysis. The CD56^dim^CD16^+^ human NK cell population was isolated via FACS sorting (BD FACSAria IIu) of cells stained with CD56-PE (Beckman Coulter, Pasadena, CA) and CD16-FITC, Clone VEP13 (Miltenyi Biotec, Bergisch Gladbach, Germany) antibodies, yielding a population that was > 98 % pure. Human NK cells were cultured in RPMI-1640 medium supplemented with 10 % heat-inactivated pooled human AB serum (HAB) (C-six Diagnostics; Germantown, WI), 100 units/mL of penicillin, 100 μg/mL of streptomycin, and 0.25 μg/mL of amphotericin B (10 % HAB medium).

### *In vitro* co-stimulation assays

For NK cell FcR activation by immobilized IgG, wells of a 96-well flat-bottom plate were coated with 100 μg/mL of polyclonal huIgG in cold PBS overnight at 4 °C. Plates then were washed, and human NK cells were plated at 2 x 10^5^ cells/well with IL-12, as previously described [9]. For NK cell FcR activation by FcγRIIIa clustering, human NK cell FcγRIIIa were cross-linked with F(ab’)_2_ fragments of monoclonal Ab (mAb) 3 g8 and goat F(ab’)_2_ anti-mouse Ig secondary Ab, in the presence of 10 ng/mL huIL-12. For FcR activation by NK cell co-culture with Ab-coated tumor cells, wells of a 96-well flat-bottom culture plate were coated with the HER2-overexpressing cell line SK-BR-3. 5 x 10^4^ tumor cells were grown to confluence overnight at 37 °C. The culture supernatant was aspirated the following day, wells were treated with 100 μg/mL trastuzumab (Herceptin™, an anti-HER2 mAb) for 1 h at 37 °C and then washed twice with warm medium to remove any unbound Ab. Purified NK cells were added at 2 x 10^5^ cells/well in the presence of 10 ng/mL rhuIL-12, as previously described [9]. Control wells contained tumor plus NK cells supplemented with medium alone, trastuzumab alone, or rhuIL-12 alone. Following activation, cell-free culture supernatants were harvested at the indicated time-points and analyzed for levels of human cytokines (IFN-γ, TNF-α, and MIP1-α) by ELISA (Endogen, Inc, Rockford, IL). Statistical analysis of ELISA cytokine levels was performed using the Student’s paired *t*-test, with *p* < 0.05 considered significant.

### Preparation of labeled RNA and microarray hybridization

Total cellular RNA from NK cells obtained from individual donors (*n =* 8) was isolated and subjected to a cleanup protocol with RNeasy mini kits (Qiagen, Valencia, CA), according to the manufacturer’s specifications. The quality of total RNA was assessed using an Agilent Bioanalyzer. First and second strand cDNA was prepared from 8 μg of total RNA, and the cDNA was subjected to *in vitro* transcription in the presence of biotinylated nucleoside triphosphates. The biotinylated cRNA was fragmented to uniform sizes and the integrity of the labeled cRNA was verified by gel electrophoresis. Affymetrix GeneChip expression array U133A was hybridized with each prepared cRNA target in duplicate, according to the manufacturer’s instructions. The raw and processed microarray data is made publicly available in GEO (GSE63038).

### Gene microarray data analysis

The DNA-Chip Analyzer (dChip) software package implementing model-based expression analysis of oligonucleotide arrays was used to obtain gene expression estimates for the samples in the study. The scanned microarray images were normalized using the invariant set scaling method at the probe level, and then model-based gene expression estimates using the Perfect Match (PM) Only Model were obtained using dChip software. When examining the reproducibility of normalized duplicates for each of the eight NK donors, significant correlation was observed between each donor duplicate (average r^2^ > 0.992, not shown).

The study was performed using a “2 x 2 factorial” complete block design with two factors, at two levels, to understand the effect of each single simulation as well as the combined simulation, considering blocking factors as “subjects” [14]. All eight subjects were treated with four treatment combinations; no stimulation (medium), FcR stimulation by immobilized IgG, IL-12 stimulation, and combined stimulation via FcR plus IL-12. An analysis of variance test was performed for the block-treatment model with the assumption that there was no interaction between blocks and treatments for each gene. Then, pairs of treatment combinations were compared by using cell mean comparison contrasts. Since six treatment comparison contrasts were performed for each gene, Bonferroni correction was applied to correct for multiple comparisons. In each comparison, gene expression values were considered to be significantly different between two treatment combinations if the p-value < 0.008, the median of fold changes of eight subjects was greater than 2 and the median of absolute differences in gene intensity of eight subjects was greater than 150. Hierarchical clustering analysis was performed using the heatmap.2 function as part of the “gplots” R package [15, 16].

Three paired comparisons were made between genes regulated in NK cells following each activation condition: 1) FcR activation by immobilized IgG vs. no stimulation (medium), 2) IL-12 vs. medium, and 3) FcR activation in the presence of IL-12 vs. medium. Venn diagrams were constructed by intersecting the set of genes up- or down-regulated by 2-fold or greater in each of the three comparisons. These diagrams were evaluated by Subject Matter Experts (SMEs) to assess the relative importance and/or impact of such up- or down-regulated genes in intersecting sets. Functional annotation (Gene Ontology) was performed for the up-regulated and down-regulated genes in each of the three comparisons using Database for Annotation, Visualization and Integrated Discovery (DAVID) software [17].

### Real-time PCR

The expression values of select genes identified via the microarray experiments were validated by Real-Time PCR. Following RNeasy purification of NK cell lysates from the immobilized IgG assay, 2 μg of total RNA was reverse transcribed and the resulting cDNA was used as a template to measure gene expression by Real-Time PCR using pre-designed primer/probe sets (Assays On Demand; Applied Biosystems, Foster City, CA) according to the manufacturer’s recommendations, as previously described [18]. Human *β*-actin (Applied Biosystems) was used as the internal control in each reaction well. Real-Time PCR data was analyzed using the ABI PRISM^®^ 7900 Sequence Detection System.

### Network driven functional enrichment analysis

#### Generating an integrated network consisting of an enriched set of functionally significant genes 

An integrated network of biomolecular features aggregated from pathway knowledge bases was created to form inductive hypotheses regarding the contribution of each differentially expressed gene in this NK cell activation study [19]. We focused our analysis on three sets of genes that were found to exhibit two-fold or greater differential expression (*p <* 0.008) when NK cells were activated in the presence of only immobilized IgG (FcR stimulus), only IL-12 (IL-12R stimulus), or both IgG and IL-12. The most common biomolecular functions identified amongst the enriched set of genes were then used as filtering criteria to aggregate additional genes with the same or similar cellular, molecular, or biological functionality. This measurement of functional enrichment indicates the proportion of genes demonstrating a specific biological function within a broader collection of genes, and serves as the basis of functional enrichment analysis workflows [20]. The resulting enriched gene sets were used to prioritize genes that are influenced upon by NK-cell activation in the presence of IL-12 and immobilized IgG. The free and open-source gene function prediction service, GeneMANIA [21], was used along with the widely used large-scale network visualization and integration tool, Cytoscape [22] to formulate and visualize the resultant integrated gene network.

#### Using Network-centric Measures to Identify Top Genes Within the Integrated Network

Once the enriched, integrated network was constructed, the next step was to identify genes that may not have been recovered by differential gene expression analysis, but might in fact be biologically interesting by virtue of functional relationships between top genes in the integrated network, regardless of whether they were identified experimentally or via computational methods. One primary criterion used for the identification of the aforementioned genes was a high degree of Betweenness Centrality (BC) relative to the rest of the integrated network [23]. BC is quantified for a particular vertex as the number of shortest paths between pairs of other vertices in the network that pass through this vertex [24]. PAJEK, a software package for manipulation and analysis of large-scale networks, was used to calculate the BC scores [25]. Additionally, the Hyperlink-Induced Topic Search (HITS) algorithm [26] was used to measure two important characteristics of an overall genomic network, including directed linkages, namely: 1) the authority of individual genes, and 2) the hub value of individual genes. A hub vertex is quantified via summing the authority vertices that it is directed to, and an authority vertex is quantified via summing the hub vertices that are directed towards it. The HITS algorithm searches for dense links between sets of highly ranked hubs pointing to sets of highly ranked authorities.

### Discovering the highest represented signaling pathways with the ingenuity pathway analysis toolkit

To complement the purely structural analysis of gene function networks, we subsequently conducted biological pathway analysis. In order to determine the top pathways represented among the genes in the integrated network described previously, the pathway analysis and visualization toolkit, Ingenuity Pathway Analysis (IPA), was used. IPA scores networks in a manner that probabilistically demonstrates that genes in the supplied data set are functionally relevant to their constituent networks. The toolkit identified several pathways that were highly relevant to the supplied gene set. The top two canonical pathways, as reported by IPA, were selected for further inspection.

## Results

### IL-12 enhances NK cell activation in response to immobilized IgG

**Fig. 2:**
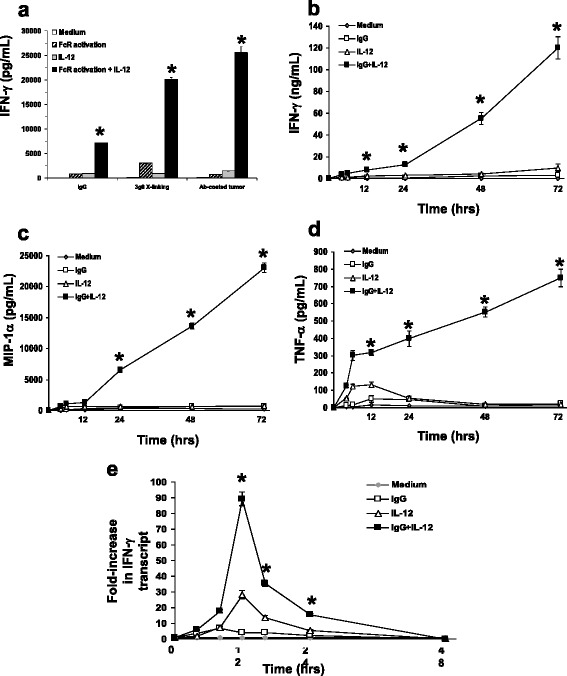
NK cells secrete high levels of immune stimulatory cytokines following FcR activation in the presence of IL-12. **a** Human NK cells were stimulated via their FcR by culture onto wells pre-coated with either huIgG or Ab-coated SK-BR-3 tumor cells, or by direct FcR cross-linking by 3 g8 Ab. IL-12 was added at a concentration of 10 ng/mL. Control wells consisted of NK cells cultured with medium alone (medium), FcR activation alone (via immobilized huIgG, Ab-coated tumor, or 3 g8 cross-linking, as indicated), or IL-12 alone (IL-12). Culture supernatants were harvested after 12 h and analyzed for IFN-γ content by ELISA. In time-course experiments, NK cells were cultured onto immobilized huIgG with IL-12 for varying times (4-72 h) and supernatants were analyzed for (**b**) IFN-γ, (**c**) MIP-1α, and (**d**) TNF-α. The means and SEM are shown with *n =* 3 for each experiment. *p < 0.001 vs. Medium, FcR activation alone, and IL-12 alone. **e** NK cells cultured in the immobilized IgG plus IL-12 condition were harvested at varying times and processed for Real-Time PCR analysis of IFN-γ transcript. Results are given as fold increase in cytokine transcript over baseline (Medium). The means and SEM are shown (*n =* 3). *p < 0.01 vs. Medium, immobilized IgG, and IL-12. Similar results were observed for gene expression of MIP-1α and TNF-α (not shown)

The aim of this study was to determine the gene expression profile of human CD56^dim^CD16^+^ NK cells following FcR activation in a pro-inflammatory environment, such as that which would occur in the presence of a cytokine like IL-12 (formerly known as NK cell stimulatory factor). Previous studies examining NK cell FcR activation have utilized NK cells cultured with low-dose IL-2 or irradiated feeder cells for maintenance of NK cell viability [27–29]. In order to ensure that the gene expression data in this study were derived from an NK cell population that had been activated through the FcR alone, we utilized an immobilized IgG assay previously established in our laboratory [9]. We have demonstrated that IL-12 signal transduction enhances the NK cell cytokine response to FcR activation *in vitro*, in murine models, and in phase I clinical trials [30–32]. Importantly, previous work also has shown that IL-12 is superior to IL-2, IL-15, IL-18, and IL-21 in triggering cytokine secretion by FcR-stimulated NK cells [33]. We therefore examined NK cell cytokine production in response to immobilized IgG in the presence or absence of IL-12 as a means of confirming the effectiveness of this stimulation strategy. Purified NK cells that were cultured for 72 h in the presence of immobilized IgG and IL-12 produced large amounts of interferon-gamma (IFN-γ), while NK cells stimulated with immobilized IgG alone or IL-12 alone produced only moderate amounts of IFN-γ. This pattern of cytokine production was similar to that produced following NK cell activation by direct FcR cross-linking or by co-culture of NK cells with trastuzumab-coated HER2/neu positive human breast cancer cells (Fig. 2a). While maximal accumulation of IFN-γ, MIP1-α, and TNF-α occurred after 72 h in culture, cytokine production could be detected within 6 h (Fig. 2b-d). Real-Time RT-PCR revealed a 20-fold increase in cytokine transcript within NK cells co-stimulated with immobilized IgG and IL-12 after 8 h in culture, with optimal transcript induction occurring at 12 h (Fig. 2e). These data confirmed that immobilized IgG represents a physiologically-relevant NK cell stimulus and indicated that this model would be adequate for assessment of global gene expression in a primary human CD56^dim^CD16^+^ NK cell population following FcR activation in the presence or absence of IL-12.

### Microarray analysis of NK cells stimulated via the FcR in the presence of IL-12

In order to begin to investigate the gene expression profiles of human CD56^dim^CD16^+^ NK cells following co-stimulation via engagement of the FcR and IL-12R, CD56^dim^CD16^+^ NK cells derived from eight healthy donors were purified and sorted by flow cytometry, plated separately in the four conditions of the immobilized IgG assay (medium, immobilized IgG, IL-12, or IgG plus IL-12) for 12 h, then harvested for RNA preparation. cRNA samples from each donor were prepared. Gene expression profiling was performed by individually hybridizing the cRNA samples to the Affymetrix GeneChip expression array U133A, which interrogates 14,500 human genes.

**Fig. 3:**
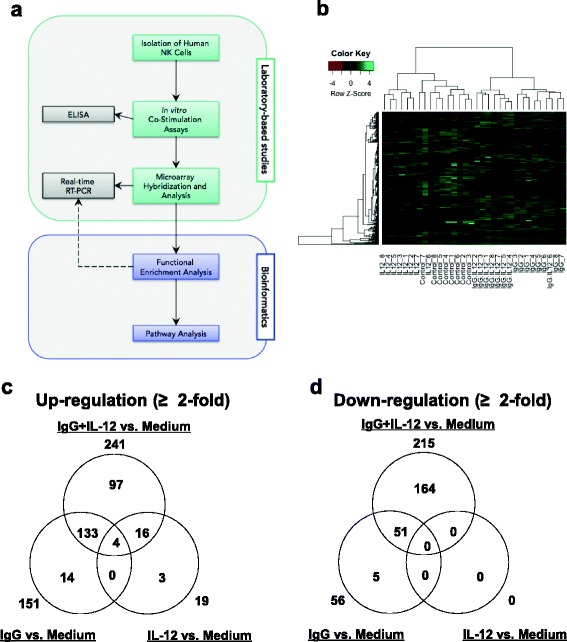
FcR activation in the presence of IL-12 results in differential expression of a unique subset of genes. **a** Human NK cells were isolated from peripheral blood and cultured with immobilized IgG, IL-12, or IgG and IL-12. Following stimulation, culture supernatants were harvested and levels of cytokines were analyzed by ELISA. Genomewide expression profiling was performed for NK cells derived from all 8 samples via microarray, and representative transcript expression levels were validated using Real-time RT-PCR. The bioinformatics portion of this study utilized a set of genes that exhibited the greatest expression level differences as reported by microarray. Functional enrichment analysis generated an integrated network of structural interactions among differentially expressed genes. Canonical pathway interactions were mapped using IPA. Select genes within top enriched GO functional categories were validated using Real-time RT-PCR (dotted line). **b** Hierarchical clustering based on the expression profile of genes that were up- or down-regulated by at least 2-fold across all samples. Each row represents relative hybridization intensities of a particular gene across different samples. Colors reflect the magnitude of relative expression of a particular gene across samples. Brighter green corresponds to higher expression and brighter red corresponds to lower expression. The organization and length of the branches in the resulting dendrogram reflect the similarity in gene expression profiles between each of the samples. The division and length of the branches within the dendrogram reflect the relative similarity in gene expression profiles between each NK cell stimulation condition. The terminal branches that are close spatially in the dendrogram represent the NK cell conditions that have the most similar gene expression patterns. Venn diagrams generated by the intersection of the list of genes (**c**) up-regulated or (**d**) down-regulated by at least 2-fold vs. stimulation with medium alone are shown

A list of differentially expressed genes among the four stimulation conditions was compiled. Initially, four sets of gene expression intensity values were generated for each NK cell donor: resting NK cells (medium), immobilized IgG-stimulated NK cells, IL-12-stimulated NK cells, and IgG plus IL-12 co-stimulated NK cells, and next, bioinformatics analyses were performed to examine the most highly represented structural and functional relationships between differentially genes (Fig. 3a). The gene expression patterns for the four stimulation conditions were organized by a hierarchical clustering algorithm of variable genes that exhibited a 2-fold or greater change in expression. Mean levels were calculated for each gene across all samples. The magnitude of expression of a particular gene relative to the calculated mean expression was reflected by use of color representation. The resultant gene expression profiles are presented in heatmap format with corresponding dendrograms for samples and genes (Fig. 3b). The organization and length of the branches in the dendrogram reflect the similarity in gene expression profiles between each of the NK cell stimulation conditions. The distance between the branch lengths (on the horizontal axis) reflects the comparative difference in gene expression profiles between each sample. Hierarchical clustering across all samples using differentially expressed genes across any of the three comparisons showed that the four main sub-groups were correctly classified into the corresponding four NK cell stimulation conditions with only 3 misclassifications (IL-12 sample 7, IL-12 sample 6 and IgG-IL-12 sample 6). Importantly, NK cells stimulated via the FcR (in the presence or absence of IL-12) exhibited gene expression patterns that were significantly different from that observed following stimulation with media or IL-12 alone. These initial observations suggested that the gene expression profile of immobilized IgG plus IL-12 stimulated NK cells was distinct from that of single-condition stimulated cells and provided the rationale to further investigate the expression profile of co-stimulated NK cells.

In order to confirm the quality of these experimental results, these data were evaluated and correlated with existing gene expression data from the literature. The gene expression levels of bonafide NK cell activation markers were found to be up-regulated upon co-stimulation of NK cells via immobilized IgG and IL-12. For example, as seen in previous reports, gene expression levels of *IFN-γ* and *granzyme B* were found to be up-regulated in response to FcR and IL-12R co-stimulation. [29, 34]. Several other genes that were up-regulated upon activation include *CKS2, CCND2, CXCL10*, and *IL-4R* [29, 35]. The NK cell receptors *2B4* and *KLRF1* were down-regulated when NK cells were co-stimulated via immobilized IgG and IL-12, which also is consistent with previously published reports [36, 37]. (See Additional file 1 for the full list of differentially regulated genes.)

Analysis of gene expression data was initiated by performing the following comparisons: 1) immobilized IgG stimulated NK cells vs. unstimulated NK cells (medium), 2) IL-12 stimulated NK cells vs. medium, and 3) IgG plus IL-12 co-stimulated NK cells vs. medium. Genes that were differentially expressed 2-fold or greater under the stimulation conditions were included in the comparisons. Additional file 1 lists genes and their change in expression calculated from these three comparisons. The total number of genes up- or down-regulated by each stimulation condition were arranged into Venn diagrams in order to globally assess those genes that were modulated across multiple stimulation conditions and those genes that were uniquely up- or down-regulated by a specific stimulus (Fig. 3c-d). The gene expression profile of immobilized IgG-stimulated NK cells revealed that there were 151 genes that were significantly up-regulated by > 2-fold (14 of those genes were up-regulated uniquely by this stimulus), while only 56 genes were down-regulated > 2-fold (5 down-regulated uniquely). IL-12 stimulation led to a substantial up-regulation of 19 genes (3 unique to this stimulus) while no genes were significantly down-regulated. *IFNG* (also referred to as IFN-γ) was found to be up-regulated 10-fold, which is consistent with previous reports describing IL-12 mediated NK cell activation [38]. Finally, 241 genes were up-regulated in response to immobilized IgG plus IL-12 co-stimulation (97 uniquely), and 215 genes were down-regulated (164 uniquely). Of note, only 4 up-regulated genes overlapped among the three comparison groups, while the majority of up-regulated genes, 133/151 and 16/19, uniquely overlapped between IgG and IgG + IL-12 and IL-12 and IgG + IL-12, respectively. Similarly, the majority of down-regulated genes in the IgG stimulation condition (51/56) was also recovered in the IgG + IL-12 condition. Taken together, these data suggest that NK cells co-stimulated via the FcR and IL-12R express a genetic profile that is unique, synergistic, and non-additive.

### Gene ontology classification of microarray data

Next, up-regulated and down-regulated genes were categorized based on their functional characteristics from available gene ontology (GO) annotations in public databases (Additional file 2a-b). Additional file 3 contains a complete list of the individual genes within each functional category. Genes that were significantly up-regulated in response to immobilized IgG and the combination of IgG plus IL-12 revealed that the following functional categories were over-represented in this list of genes relative to all annotated genes on the array: response to biotic stimuli, chemotaxis, NK cell mediated cytotoxicity, and promoters of apoptosis. The only GO term significantly represented by the IL-12 condition was JAK-STAT signaling, which is the predominant cytokine-associated cell-signaling pathway [39]. Similarly, statistical analyses of the genes that were down-regulated showed that the following functional categories were over-represented in IgG plus IL-12 NK cells: cell membrane-linked signal transduction, cell adhesion, response to biotic stimuli, cell cycle control, phosphorylation, and apoptosis.

### Genes uniquely regulated in NK cells following FcR activation in the presence of IL-12

**Fig. 4:**
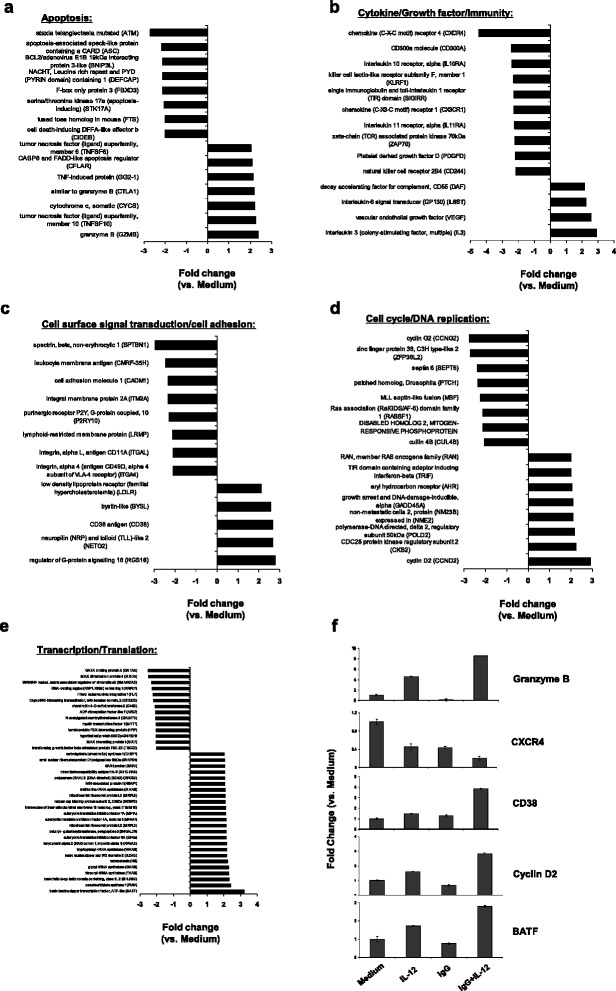
Genes uniquely regulated in NK cells following FcR activation in the presence of IL-12 grouped by function. Gene expression values of genes differentially expressed by at least 2-fold by NK cells stimulated by immobilized IgG plus IL-12 vs. those stimulated by Medium. Bars represent fold change of the mRNA level of a particular gene when comparing the subpopulations. Positive values indicate that the transcript was more abundant in the stimulated cells and negative values indicate that the gene was down-regulated in the stimulated cells. Genes were grouped according to their presumed function (**a**-**e**) based on information available in public databases or in the literature (see Materials and Methods). **f** Validation of microarray gene expression estimates by Real-Time PCR. Shown is fold change in gene expression (vs. no stimulation) within NK cells following FcR activation (IgG), IL-12 stimulation (IL-12), or FcR activation in the presence of IL-12 (IgG plus IL-12). Representative data from a single donor out of three examined is shown for each gene listed on the right

Given that the dual stimulation condition might best represent how NK cells respond upon encountering antibody-coated targets within an inflammatory environment, we subsequently examined those genes that were uniquely regulated by FcR activation in the presence of IL-12. The expression profile of the 97 uniquely up-regulated and 164 uniquely down-regulated genes in co-stimulated NK cells was analyzed by functional annotation of GO terms (DAVID). Functional categories and their constituent genes are depicted in Fig. 4a-e. Within the apoptosis category, genes encoding endogenous pro-apoptotic molecules such as *ASC* (apoptosis-associated speck-like protein containing a CARD), *BNIP3L* (BCL2/adenovirus E1B 19 kDa interacting protein 3-like), *STK17A* (serine/threonine kinase 17a apoptosis inducing) and *CIDEB* (cell death-inducing DFFA-like effector b) were all negatively regulated. This suggests enhanced pro-survival signaling within the co-stimulated NK cells as compared to those cells activated by a single stimulus. Also, consistent with the known cytotoxic capabilities of activated CD56^dim^CD16^+^ NK cells, our analysis revealed several genes encoding secreted or cell-surface cytolytic molecules in the apoptosis grouping that were uniquely up-regulated in co-stimulated NK cells; e.g., *granzyme B, cytochrome c*, and multiple TNF superfamily ligands (Fig. 4a). In the cytokines and growth factors category, *CXCR4* (chemokine [C-X-C motif] receptor 4), *IL10RA* (interleukin 10 receptor alpha), *CX3CR1* (chemokine [C-X3-C motif] receptor 1) and *IL11RA* (interleukin 11 receptor alpha) were down-regulated 3–5 fold (Fig. 4b). In the same category, three inhibitory NK cell receptors (NKRs): *CD244* (natural killer receptor 2B4), *CD300a*, and *KLRF1* (killer cell lectin-like receptor subfamily F, member 1) were all uniquely down-regulated, confirming the activated state of the stimulated NK cells. The cytokine *IL-3* was uniquely up-regulated, which may suggest a unique mechanism by which activated NK cells promote the development and proliferation of other hematopoietic cells. The cell surface signal transduction and cell adhesion molecules *CADM1* (cell adhesion molecule 1), *SPTBN1* (spectrin, beta, non-erythrocytic 1), *ITM2A* (integral membrane protein 2A) and *P2RY10* (purinergic receptor P2Y, G-protein coupled, 10) were uniquely down-regulated, and *CD38* and *RGS16* (regulator of G-protein signaling 16) were uniquely up-regulated (Fig. 4c). In the category cell cycle and DNA replication, uniquely down-regulated genes included *CCNG2* (cyclin G2), *ZFP36L2* (zinc finger protein 36, C3H type-like 2), *SEPT6* (septin 6). In the same category *CCND2* (cyclin D2), *CKS2* (CDC28 protein kinase regulatory subunit 2) were uniquely up-regulated (Fig. 4d). Fig. 4e shows the expression profiles of select genes within the transcription and translation category. The uniquely down-regulated genes included several DNA binding proteins, e.g., *GATA3* (GATA binding protein 3), *CITED2* (Cbp/p300-interacting transactivator, with terminal domain, 2) and *HPIP* (hematopoietic PBX-interacting protein). The uniquely up-regulated genes included *BATF* (basic leucine zipper transcription factor) and several translation initiation factors (e.g., *EIF1A, EIF4A1,* and *EIF5a*).

Real-Time PCR verified the predicted changes in expression of representative genes in response to NK cell co-stimulation via immobilized IgG and IL-12 within enriched functional categories (Fig. 4f). Granzyme B may be released in cytoplasmic granules from cytotoxic T cells and NK cells to induce apoptosis of target cells, and RT-PCR confirmed that this gene is up-regulated following co-stimulation of NK cells (Figs. 4a and f). In the cytokine and growth factor category, *CXCR4* down-regulation was also validated by Real-Time PCR (Figs. 4b and f). CXCR4 has been reported to be expressed on NK cells and is involved in the migration of NK cells in response to CXCR4 ligand [40]. In the category cell surface signal transduction, the gene *CD38* was up-regulated approximately 4-fold (Figs. 4c and f). CD38 has been shown to associate with the FcR to activate signal transduction pathways, which includes calcium fluxes, tyrosine phosphorylation of ZAP70 and mitogen-activated protein kinase, and secretion of IFN-γ and GM-CSF [41, 42]. *Cyclin D2*, which belongs to the cell cycle/DNA replication category, was confirmed to be up-regulated 3-fold (Figs. 4d and f). In the category transcription and translation, *BATF* was found to be up-regulated 2.8-fold (Figs. 4e and f). In addition to Real-Time PCR analyses, we further incorporated bioinformatics-based approaches to gain insight regarding the mechanism of NK cell activation following FcR stimulation in the presence or absence of IL-12.

### Leveraging network-centric measures to discover novel genes

It is recognized that significant alterations in individual gene expression may not comprehensively reflect the biological response of NK cells to FcR and IL-12R stimulation. Thus, we constructed a functionally enriched, integrated network connecting the differentially expressed genes identified in this study with other genes in the same or similar functional categories via GeneMANIA in order to: i) elucidate the biological context of gene expression changes of immobilized IgG and IL-12 stimulated NK cells and ii) identify additional key genes that might be involved in NK cell activation. A subset of this network including the most highly connected genes is shown in Additional file 4. Table 1 lists the top twenty genes in the network ranked according to their betweenness centrality (BC) measures. BC is an indicator of the primary “bridges” between functionally segregated clusters within a gene network (see Methods). This specific relative ranking was selected as one of the criteria to analyze the network since gene-to-gene functional linkages were found to be mostly unidirectional. That is, if a gene influenced another gene via a given functional linkage, then the influenced gene would rarely reciprocate along the same functional linkage. The *centrality* of a node reveals its connectivity with other nodes, or genes, in an integrated network. Of note, the IL-21 receptor, *IL21R*, was found to exhibit the highest BC score in the integrated network. This indicates that the IL-21 receptor serves as a critical link between other important genes and relationships within the activated NK cell gene network. Importantly, 6 out of the 20 highly ranked BC genes (*CCR5, CXCR6, IL2RB, IRF1, STAT4* and *TBX21*) were not recovered in our filtering criteria to identify differentially expressed genes in the original microarray experiments and were exclusively identified via mapping functional linkages as described in the Methods section.

**Table 1:** List of most highly ranked genes based on Betweenness Centrality (BC) score

Rank	Gene	Betweenness Centrality Score
1	IL21R	0.0921779
2	FASLG	0.0612643
3	IRF4	0.0545417
4	STAT4	0.0455257
5	TNFRSF4	0.0438375
6	IL2RB	0.0244015
7	CCR5	0.0233243
8	P2RX5	0.0203641
9	SLC7A5	0.0195173
10	BATF3	0.0192476
11	TNFRSF9	0.0188821
12	TBX21	0.0176988
13	IFNG	0.0171686
14	IRF1	0.0171325
15	ACAP1	0.0170313
16	CXCR6	0.0149211
17	TRIB1	0.0146721
18	EBI3	0.0141001
19	NFKB2	0.0134729
20	EIF4A1	0.0132968

BC scores were calculated via the PAJEK algorithm (22), and are presented in decreasing order within the integrated network. A higher BC score indicates that a given gene is highly interconnected with other genes and gene products

In order to illustrate a deeper level of network connectivity analysis beyond betweenness centrality, including connection cardinality, we further analyzed genes in the network according to authority and hub metrics. Table 2a classifies the highest ranked genes according to their authority measures (*n =* 9), while Table 2b includes a set of genes ranked according to their hub measures (*n =* 9). Using the HITS algorithm, a node achieves a high authority measure when several important nodes within the integrated network point to it. In this study, a high authority measure indicates a gene that is a suitable indicator of the functional patterns in its neighborhood within the gene network. In contrast, a high hub measure is an indicator of the direct connections a node makes with other nodes in the integrated network. If a node with a high hub measure is removed from the network, the entire network will be disrupted, and previously connected sub-networks will fragment into unconnected gene clusters. In order to obtain numerical scores, authority nodes are quantified via summing the hub nodes that are directed towards them, and hub nodes are quantified via summing the authority nodes that it are directed towards them. *NLRP1* was the highest ranked authority gene, followed by *BIRC3* and *EIF4A1*. In regards to important hub genes, *CASP1* was found to be the most important followed by *FASLG* and *TRAF1*. Of note, all of the authority genes were identified in the original microarray differential expression analyses. Interestingly, nearly 45 % (4 out of 9) hub genes were exclusively recovered via computational means and mapping functional linkages. These four genes include *NFKBIA*, *PTPRC*, *TNFRSF1B* and the top-ranked hub gene, *CASP1*. Further, no individual gene was identified as a combined highly ranked BC, authority, and hub gene. Only 1 out of the 9 hub and 9 authority genes was found to be common between the two groups: namely *FAS*. Similarly, only *FASLG* was identified as both a hub and BC gene. A slightly higher proportion of authority genes (3/9) were found to be BC genes, and vice versa (3/20 BC genes): *EIF4A1, TNFRSF9*, and *IFNG*.

**Table 2:** List of most highly ranked genes based on Authority or Hub scores

A	
Rank	Authorities
1	NLRP1
2	BIRC3
3	EIF4A1
4	FAS
5	CFLAR
6	TNFRSF9
7	PTPRCAP
8	EGR3
9	IFNG
B	
Rank	Hubs
1	CASP1
2	FASLG
3	TRAF1
4	NFKB1
5	NFKBIA
6	TNFRSF1B
7	FAS
8	IL11RA
9	PTPRC

The HITS algorithm was used to measure two important characteristics of an overall genomic network: the authority of individual genes, which may be interpreted as a surrogate marker for the relative “importance” of those genes to the overall function of the network (Table 2a); and the hub value of individual genes, which is a surrogate marker for the relative “impact” of that gene on the interconnections or relationships between genes that make up that same network (Table 2b). Authority and Hub scores are presented in decreasing order within the integrated network

### Discovering the highest represented pathways

**Fig. 5:**
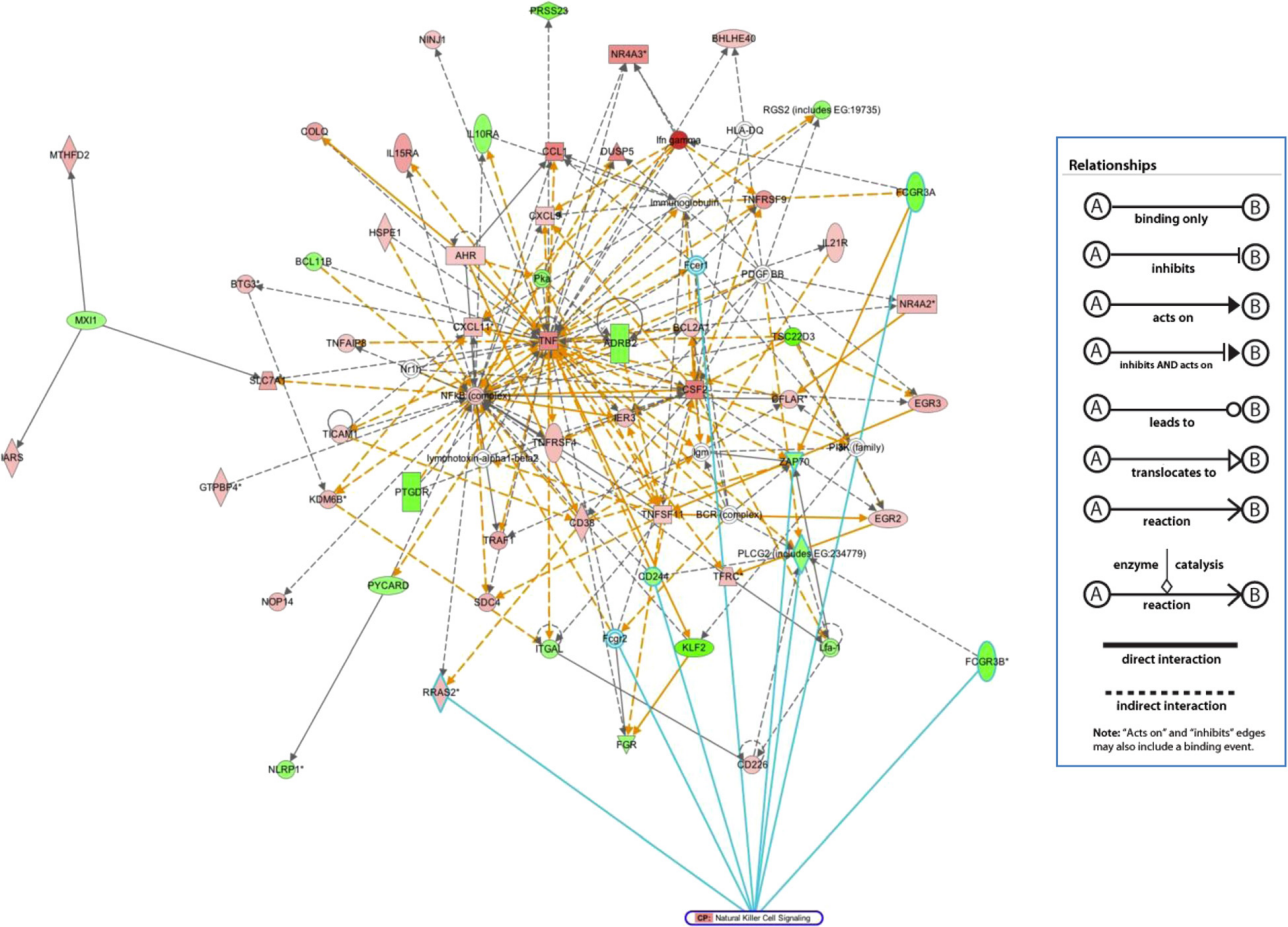
An integrated visualization of the top two networks generated by IPA. The connections between genes in the NK cell signaling pathways have been highlighted using blue edges. Red nodes represent up-regulated genes, and green nodes represent down-regulated genes identified in our differential expression analysis (based on NK cells dual-stimulated via IgG and IL-12). The darker the node color, the more extreme (high or low) the up- or down-regulation of the respective gene. White nodes represent genes that were not originally identified in our microarray analysis but are connected to other genes within the enriched network. Blue nodes represent genes from canonical pathways. Relationship types are outlined in the figure legend

We next used Ingenuity Pathway Analysis (IPA) to illustrate the most common canonical pathways associated with the genes that were differentially expressed (2-fold or greater regulation) in NK cells stimulated by immobilized IgG plus IL-12 as well as those contained in the enriched integrated network for this stimulation condition. The top two IPA pathway networks are shown in Fig. 5. The top two networks were chosen so that the resultant integrated network was not too dense to be visualized as a two-dimensional image. Table 3 lists constituent genes of the top two pathways, where the differentially expressed genes identified in the original microarray analysis are highly represented. Future functional studies of the key genes regulating NK cell signaling pathways identified in these analyses may unveil previously unknown drivers of NK cell function in the setting of monoclonal antibody therapy for cancer.

**Table 3:** Top two networks along with their constituent genes and associated canonical scores as calculated by IPA.

ID	Molecules in the Network	IPA Score	Top Functions
1	**ADRB2**, **BCL11B**, **BHLHEA40**, **BTG3**, **CD38**, **COLQ**, **CSF2**, **DUSP5**, **EGR2**, **EGR3**, Fcgr2, **FCGR3A**, **FGR**, HLA-DQ, **HSPE1**, **IARS**, **IER3**, Ifn gamma, **IL21R**, **KDM68**, lymphotoxin-alpha1-beta2, **MTHFD2**, **MXI1**, **NINJ1**, Nr1h, **NR4A2**, **NR4A3**, PDGF BB, Pka, **PRSS23**, **PTGDR**, **RGS2** (**includes EG:19735**), **SDC4**, **SLC7A1**, **TNF**	37	Connective Tissue Disorders, Immunological Disease, Inflammatory Disease
2	**AHR**, **BCL2A1**, BCR (complex), **CCL1**, **CD226**, **CD244**, **CFLAR**, **CXCL9**, **CXCL11**, Fcer1, **FCGR3B**, **GTPBP4**, Igm, **IL10RA**, **IL15RA**, Immunoglobulin, **ITGAL**, **KLF2**, Lfa-1, NFkB (complex), **NLRP1**, **NOP14**, PI3K (family), **PLCG2** (**includes EG:234779**), **PYCARD**, **RRAS2**, **TFRC**, **TICAM1**, **TNFAIP8**, **TNFRSF4**, **TNFRSF9**, **TNFSF11**, **TRAF1**, **TSC22D3**, **ZAP70**	37	Cellular Development, Cellular Growth and Proliferation, Hematological System Development and Function

The gene names in bold text indicate those genes that were included in the original microarray data set for NK cells stimulated via IgG and IL-12. The assertion “top two” indicates that the maximal number of genes from the integrated network belonged to these two pathways according to the IPA analysis tool

Together, results derived from both canonical pathway and topological network analyses provide insight into the diverse, interwoven network of genes involved in NK cell activation. The systems-level observations generated in this study may serve as a basis for identification of important candidate genes to be investigated in future functional studies and subsequently, serve as targets for diagnostic or therapeutic interventions.

## Discussion

The major goal of this study was to gain insight into the function of NK cells upon encounters with Ab-coated targets in the presence of locally secreted cytokines from both an immunological and bioinformatics-based perspective. In the present study, we utilized oligonucleotide microarrays to evaluate the gene expression profile of NK cells following FcR activation in the presence of the powerful pro-inflammatory cytokine, IL-12. We further employed several systems-level analyses, including functional enrichment as well as network centrality and pathway analysis, in order to determine key genes and biological functions influencing the responses of NK cells encountering Ab-coated target cells in an inflammatory environment. The most common biomolecular functions identified amongst the differentially expressed genes were utilized to aggregate additional genes with the same or similar cellular, molecular, or biological functionality into an integrated network. Furthermore, network analysis metrics, including betweenness centrality, authority, and hub measures were employed to rank the relative importance of genes in the overall integrated network. To our knowledge, this is the first application of sophisticated network-level analyses to describe the genetic profile of a clinically relevant population of activated NK cells. We report herein the gene expression profile induced within FcR and cytokine stimulated NK cells and highlight potential key regulators of NK cell activity in inflammatory settings.

Several unique genes in the combined immobilized IgG plus IL-12 stimulation condition exhibited significance in both the microarray data set as well as the enriched gene network generated in the bioinformatics-driven portion of this study. Select members of the Tumor Necrosis Factor Receptor Superfamily (TNFRSF) were found to be significantly up-regulated in the microarray gene expression studies and were identified as key entities in the overall enriched network (*TNFRSF4*: BC Rank 5, *TNFRSF9:* BC Rank 11 and Authority Rank 6, and *TNFRSF1B*: Hub Rank 6). Accordingly, TNF-α induction in immobilized IgG plus IL-12 activated NK cells was confirmed via enzyme-linked immunosorbent assays (ELISA) as well as RT-PCR studies. Moreover, the Basic leucine zipper transcription factor, ATF-like (*BATF*, BC Rank 10) was the most highly up-regulated gene in the Transcription/Translation category of the microarray studies. This gene has been shown to interact with Interferon Regulatory Factor 4 (*IRF4*, BC Rank 3 in the enriched network generated in this study) to promote transcriptional activation in T cells, support development of dendritic cells, and to aid in the immune response against invading pathogens [43, 44]. The results in this study may indicate a potential role for *BATF* and *IRF4* interactions in the NK cell response to immune-based therapies. Also, recent studies have examined the role of the Aryl Hydrocarbon Receptor (AHR) in NK cell development [45]. It has been suggested that AHR influences the expression of the transcription factor T-Box Protein 21 (TBX21) and has downstream effects on NK cell IFN-γ production. In this study, IFN-γ production was significantly increased in FcR and IL-12R activated NK cells, and this gene exhibited a BC Rank 13 and Authority Rank 9 in the enriched gene network. Moreover, *AHR* expression was up-regulated 2-fold in the microarray data set, and *TBX21* exhibited a BC Rank 12 in the enriched gene network. The importance of *AHR*, *TBX21*, and *IFNG* were highlighted in both laboratory- and bioinformatics-based portions of this study, supporting further investigation into their interactions and potential role in the function of activated NK cells. In summary, these findings indicate that genes identified using genome-wide profiling and those that were prioritized via computational functional enrichment and pathway analyses may serve as interesting targets for future studies to better understand the mechanisms underlying NK cell activation.

Interestingly, bioinformatics-based analyses revealed that the *IL21R* exhibited the highest BC score within the activated NK cell gene network, indicating its importance within the genetic framework governing NK cell anti-tumor activity. In the laboratory-based studies, evaluation of *IL21R* expression by Real-Time PCR revealed an intriguing differential regulation that may explain the mechanisms utilized by NK cells upon encounters with Ab-coated targets in different environments. For example, expression of the *IL21R* was significantly enhanced in response to FcR activation alone, but neither enhanced nor inhibited in response to FcR activation in the presence of IL-12. Previous reports have shown that IL-21 enhances CD56^dim^CD16^+^ NK cell cytotoxicity and cytokine production in response to Ab-coated targets [46, 47]. Hence, up-regulation of the *IL21R* upon encounters with Ab-coated targets alone may reflect an endogenous mechanism that allows for rapid enhancement of NK cell activity responsible for further inflammation and recruitment of the adaptive immune response. As a key player within the integrated gene network, additional investigation is essential in order to better understand the complex role of IL-21 and its receptor on NK cell anti-tumor activity.

Further, given that immune cell crosstalk plays a critical role in the anti-tumor immune response, we were interested in the regulation of genes that may facilitate cellular interactions between activated NK cells and other immune cells within the tumor microenvironment. In particular, myeloid-derived suppressor cells (MDSCs) increasingly have been recognized as major players in suppression of the anti-tumor immune response. While several manuscripts have highlighted the importance of interactions between T cells and MDSCs, less is known about the relationship between NK cells and MDSCs [48, 49]. The current study provides several interesting observations suggesting the potential for crosstalk between activated NK cells and MDSCs. Following stimulation of the FcR and IL-12R, NK cell expression of the IL-10 receptor (*IL10RA*) was significantly down-regulated according to the microarray and bioinformatics-based analyses. This is of interest, as several reports have shown that MDSCs produce IL-10, and IL-10 also has been shown to promote NK cell proliferation and cytotoxic activity against tumor cells [50–53]. In addition, dual stimulated NK cells exhibited increased expression of *CSF2* (also known as GM-CSF) and *TNF*. Both of these cytokines have been demonstrated to drive the accumulation and suppressive function of MDSCs in multiple tumor models [54, 55]. *TNFRSF9* was shown to be an important player in both the microarray data set and in various bioinformatics-based analyses as well. TNFRSF9 associates with 41BB to form CD137, a T cell and NK cell co-stimulatory molecule. Signaling via CD137 has been shown to suppress CD4^+^ T cell activation, stimulate CD8^+^ T cell activity and increase proliferation of CD11b^+^Gr-1^+^ myeloid derived suppressor cells (MDSCs) [56]. Notably, recent studies have demonstrated increased CD137 expression on NK cells following interactions with mAb-coated tumor cells [57]. NK cell anti-tumor activity therefore may be enhanced via combination therapy including a tumor antigen-specific mAb and an agonistic anti-CD137 mAb. In addition, it may be important to examine the impact of anti-CD137 mAb therapy on inhibitory MDSC in future studies. Lastly, we observed an increase in *CCL1* expression in dual stimulated NK cells. Studies have shown that MDSCs from patients with urothelial and renal cell carcinoma express CCR8 (the CCL1 receptor), and tumor-derived CCL1 promotes the accumulation of MDSCs in both the peripheral blood and tumor tissue [58]. While identifying potential interactions between NK cells and MDSCs was not the primary objective of this study, our observations suggest that further investigation to better understand NK cell and MDSC crosstalk will yield informative results regarding the immune response to monoclonal antibody therapy for cancer.

Network-based approaches have been successfully applied in a variety of immune cell contexts. In a recent study comparing the transcriptional responses of NK cells activated via *P. falciparum* laboratory strain 3D7 and NK cells treated with IL-12 and IL-18, the authors used microarray and networks analysis to identify distinct genes and pathways involved in these responses including TREM1 signaling and protein ubiquitination processes [59]. In another study analyzing gene expression signatures using Ingenuity database information and Bayesian inference in the context of T cell activation, Jagged1 was shown to modulate T cell activation in PBMCs from multiple sclerosis patients [60]. The authors further showed that a Jagged1 agonist ameliorated the disease course in a mouse model of autoimmune encephalomyelitis. Additional applied immunoinformatics frameworks include the statistically powerful weighted gene co-expression network analysis (WGCNA), whereby novel biological modules and pathways may be discovered [61], as well as ontology-based methods to define novel biomarkers and infer biological functions [62].

Our immunological evaluation of activated NK cells could be limited in the current study because only one type of co-stimulation condition, immobilized IgG and IL-12, was examined. Of note, as shown in Fig. 3a, NK cell IFN-γ production was greater in response to co-culture with trastuzumab-coated cells and IL-12 compared to that of immobilized IgG and IL-12 stimulated cells. This finding could indicate that a potentially different stimulatory environment may be encountered by NK cells interacting with Ab-coated tumor cells as compared to those that engage only immobilized IgG. Further, incorporating multiple NK cell stimulation strategies may more effectively represent the complex relationships among other cytokines and NK cell activity. For instance, Smith *et al.* performed a small-scale study of global gene expression changes in NK cells co-stimulated by IL-2, IL-12, and IL-18 compared to unstimulated NK cells [63]. Like our study, the authors found that several effector cytokines, including *IFNG* as well as various TNF family members, were transcriptionally up-regulated in response to co-stimulation. Of the top 150 genes differentially expressed in the Smith *et al.* study, nearly 10 % were sequence-specific DNA-binding transcription factors, which is consistent with the central role of nuclear transcription factor responses in activated NK cells demonstrated in this study. Though our *in vitro* validation of microarray gene expression estimates by Real-Time PCR was somewhat limited in scope, future studies will include additional functional analyses of high-yield gene targets based on this initial genome-wide investigation. Also, utilizing heuristically defined thresholds (>2-fold changes in gene expression) to define significance may have limited our ability to recover genes that were more subtly expressed individually, but in combination with other genes, may represent important regulators of NK cell activation. Further interrogation of co-expression modules and integration of additional canonical knowledge and experimental evidence will advance our current findings. An important resource for future integrative genomics studies is The Immunological Genome Project (ImmGen) database of murine-derived gene expression and regulatory networks of all immune cells [64]. For example, Xue *et al.* conducted transcriptome and network-based analyses of human macrophage activation and subsequently integrated their data with core signatures of dendritic cells and macrophages contained within ImmGen. In doing so, they were able to classify several conserved genes involved in regulation of macrophage activation [65]. A preliminary analysis of ImmGen NK cell whole-genome microarray data sets demonstrated a unique transcriptional relationship between resting NK cells and T cells and may serve as a valuable resource for future studies investigating the global molecular aspects underlying NK cell function [66].

## Conclusions

This study has combined bioinformatics-driven network analyses with immunological laboratory-based studies to identify important genes modulating NK cell function following physiologically relevant activation. By utilizing an integrated approach that brings together multiple network-level analyses, we have created a compendium of important genes and pathways for future study. The results presented herein highlight the involvement of a number of genes simultaneously regulated within NK cells following cytokine and FcR activation and suggest unique mechanisms by which CD56^dim^CD16^+^ NK cells respond to Ab-coated targets under pro-inflammatory conditions. It is our hope that this study may serve as the first step towards a powerful mechanism for evaluating genetic functionality in the context of a multi-dimensional, multi-disciplinary approach to understanding NK cell function.

## Availability of supporting data

The microarray dataset may be accessed via the following Gene Expression Omnibus (GEO) link (accession number GSE63038): http://www.ncbi.nlm.nih.gov/geo/query/acc.cgi?acc=GSE63038.

## Additional files

Additional file 1:
**Supplemental Table** **1** **provides a full list of differentially expressed (2-fold or greater) genes from the microarray studies and their change in expression calculated from the following comparisons: 1) immobilized IgG stimulated NK cells vs. unstimulated NK cells (medium), 2) IL-12 stimulated NK cells vs. medium, and 3) IgG plus IL-12 co-stimulated NK cells vs. medium.** (XLS 125 kb) Additional file 2:
**2a and 2b are entitled Functional analysis of the genes regulated in NK cells following FcR activation, IL-12 stimulation or the combination reveals distinct functional grouping in each gene list (DAVID).** These figures represent the functional characteristics of up- (2a) or down-regulated (2b) genes from available gene ontology (GO) annotations in public databases. (PPT 159 kb) Additional file 3:
**Supplemental Table** **2** **contains a complete list of the individual genes within each functional category.** (XLS 45 kb) Additional file 4:
** A sub-network among 33 unique genes representing the hub, authority and high BC ranked vertices from the overall integrated network.** (PPT 407 kb)

## Abbreviations

NK: Natural killer
ADCC: Antibody-dependent cell-mediated cytotoxicity
PBMC: Peripheral blood mononuclear cell
mAb: Monoclonal antibody
IgG: Immunoglobulin G antibody
DC: Dendritic cell
MDSC: Myeloid-derived suppressor cell
NKR: NK cell receptor
GO: Gene ontology
IPA: Ingenuity Pathway Analysis
BC: Betweenness Centrality
GSEA: Gene Set Enrichment Analysis
