# Supplementary material for: Gene expression profiling of the human natural killer cell response to Fc receptor activation: unique enhancement in the presence of interleukin-12
Source: BMC Med Genomics. 2015 Oct 15;8:66. doi: 10.1186/s12920-015-0142-9 (PMC4608307; doi:10.1186/s12920-015-0142-9)
Supplement: Additional file 2: — 2a and 2b are entitled Functional analysis of the genes regulated in NK cells following FcR activation, IL-12 stimulation or the combination reveals distinct functional grouping in each gene list (DAVID). These figures represent the functional characteristics of up- (2a) or down-regulated (2b) genes from available gene ontology (GO) annotations in public databases. (PPT 159 kb) [file 12920_2015_142_MOESM2_ESM.ppt]

## Slide 1
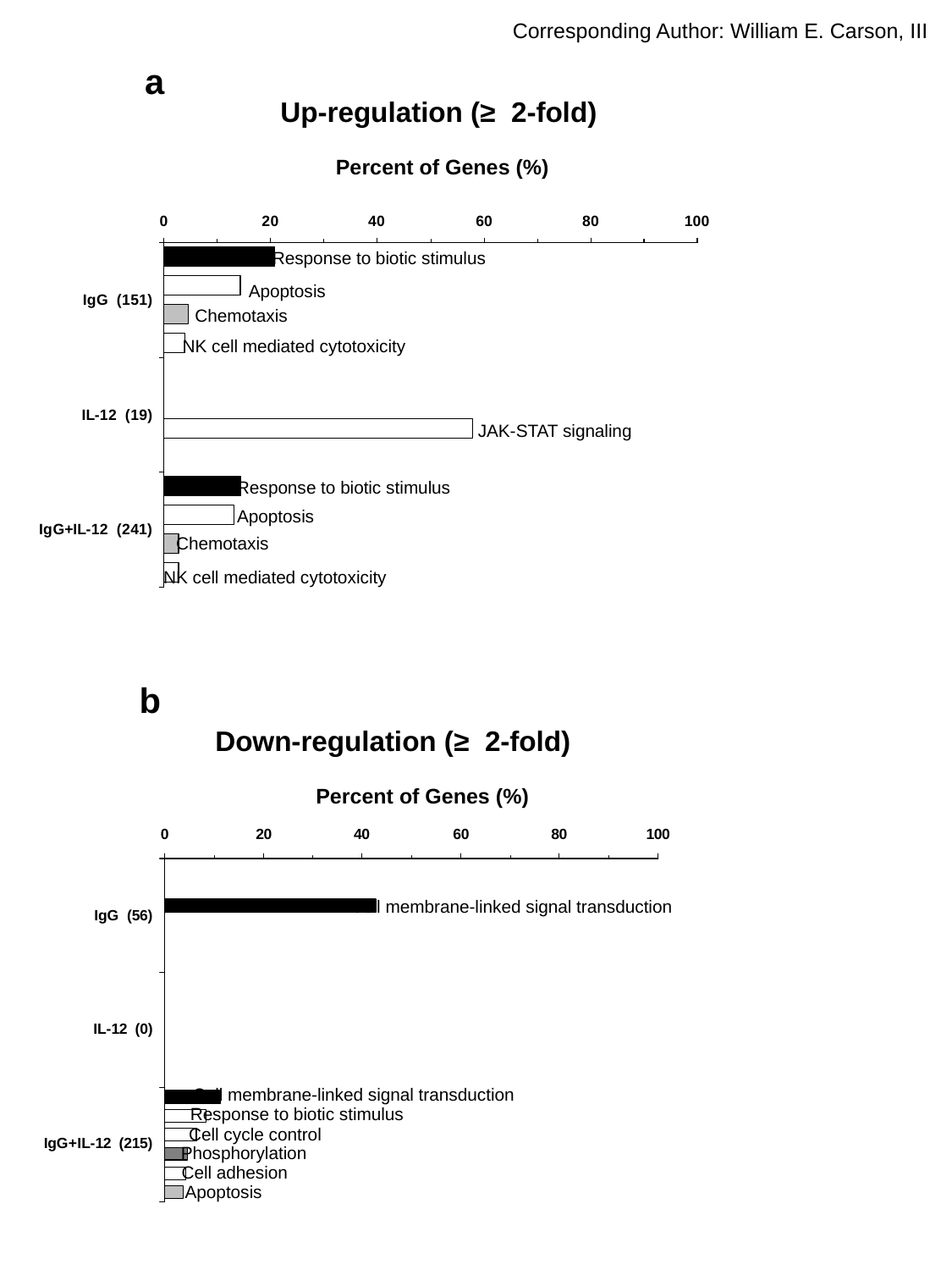

Corresponding Author: William E. Carson, III
a
Up-regulation (≥ 2-fold)
Percent of Genes (%)
Response to biotic stimulus
Apoptosis
Chemotaxis
NK cell mediated cytotoxicity
JAK-STAT signaling
Response to biotic stimulus
Apoptosis
Chemotaxis
NK cell mediated cytotoxicity
b
Down-regulation (≥ 2-fold)
Percent of Genes (%)
Cell membrane-linked signal transduction
Cell membrane-linked signal transduction
Response to biotic stimulus
Cell cycle control
Phosphorylation
Cell adhesion
Apoptosis
