# Supplementary figures and images for: Gene expression profiling of the human natural killer cell response to Fc receptor activation: unique enhancement in the presence of interleukin-12
Source: BMC Med Genomics. 2015 Oct 15;8:66. doi: 10.1186/s12920-015-0142-9 (PMC4608307; doi:10.1186/s12920-015-0142-9)

## Slide 1
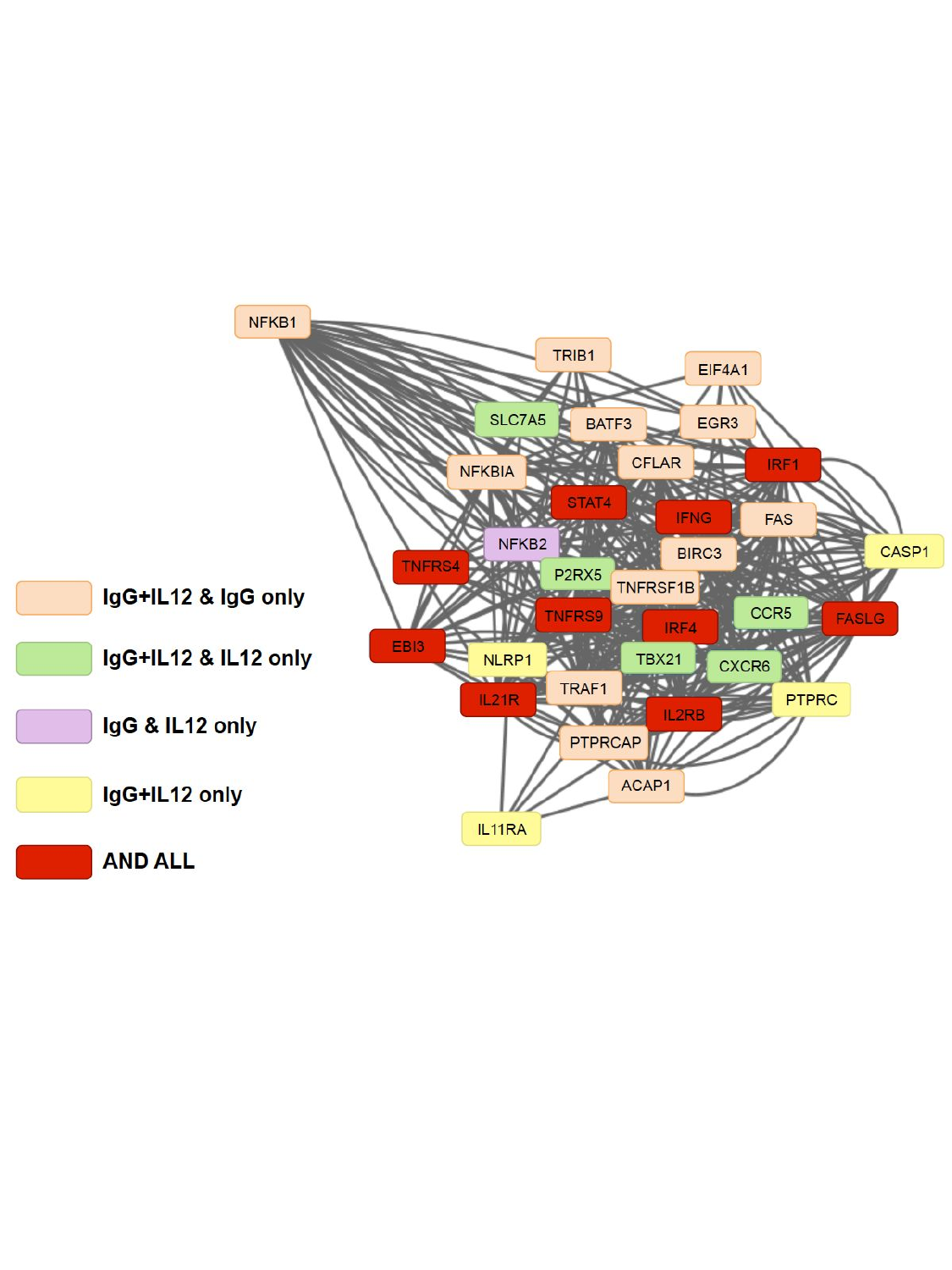

Supplement: Additional file 4: — A sub-network among 33 unique genes representing the hub, authority and high BC ranked vertices from the overall integrated network. (PPT 407 kb) [file 12920_2015_142_MOESM4_ESM.ppt]
